# Influence of La_2_O_3_ Addition on CuO/CeO_2_ Catalysts for the Water–Gas Shift Reaction

**DOI:** 10.1021/acs.langmuir.4c03606

**Published:** 2025-01-30

**Authors:** Tatiana de Freitas Silva, José Mansur Assaf, Alessandra Fonseca Lucrédio, Cristhiane Guimarães Maciel Reis, Karina Arruda Almeida

**Affiliations:** † Federal University of São Carlos, São Carlos-SP 13565-905, Brazil; ‡ São Paulo State University, São Carlos-SP 13566-590, Brazil; § Federal University of Itajubá, Itajubá-MG 37500-903, Brazil; ∥ Federal University of São João del-Rei, Sete Lagoas-MG, 35701-970, Brazil

## Abstract

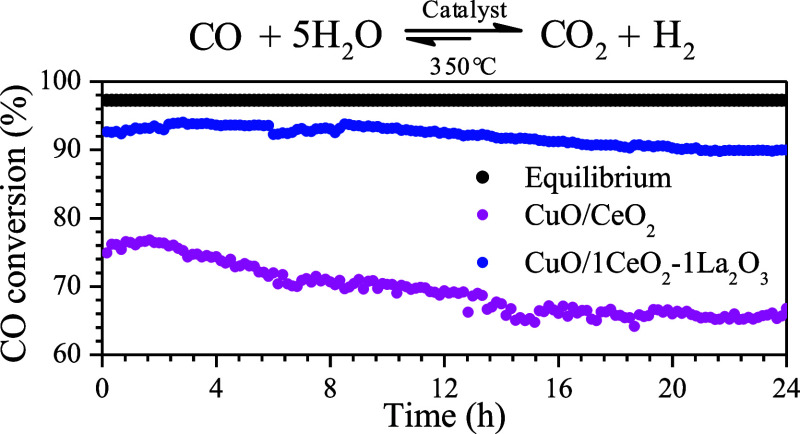

CuO/CeO_2_ and CuO/*x*CeO_2_-*y*La_2_O_3_ catalysts, synthesized with varying CeO_2_ and La_2_O_3_ molar ratios (1:1, 1:2, and 2:1), were prepared via the hydrothermal method and tested in the water–gas shift reaction (150–350 °C). La_2_O_3_ addition altered structural properties, reducing surface area and copper dispersion. XANES and in situ XRD confirmed metallic Cu species during reduction and reaction. Catalytic tests revealed CuO/CeO_2_ excelled at low temperatures, while La_2_O_3_-doped catalysts, especially CuO/1CeO_2_-1 La_2_O_3_, performed better at 250–350 °C. Stability tests showed La_2_O_3_-doped catalysts resisted deactivation and sintering better than CuO/CeO_2_, even with CO_2_ and H_2_ in the feed.

## 1 Introduction

Hydrogen is considered the most suitable alternative energy to replace conventional fossil fuels due to its renewability and environmentally friendly properties. Another advantage is that hydrogen production can be done from both renewable and nonrenewable sources.^1,2^ Current hydrogen production, however, is far from renewable; it depends almost entirely on the reforming and gasification of fossil hydrocarbon sources, such as natural gas (76%) and coal (23%).^3,4^ Alternative hydrogen production technologies, such as water electrolysis, are being researched. However, these technologies are viable only if the energy and raw materials are largely supplied from renewable sources. However, most renewable sources for electricity generation, such as wind energy and solar radiation, are inherently characterized by high volatility. Consequently, it is often necessary to use nonrenewable energy sources together.^4^

The cost of hydrogen production via water electrolysis is quite high compared to steam reforming, making steam reforming the most commonly applied process for hydrogen production.^4,5^ Steam reforming can be used with many different feedstocks, including methane, ethane, methanol, ethanol, acetone, and higher hydrocarbons. A great deal of attention has been focused on methane due to favorable byproduct formation as compared with other feeds. The disadvantage of this process lies in the hydrogen impurity produced, which contains approximately 1 to 10% carbon monoxide (CO).^3^ H_2_ stream with CO concentrations greater than 100 ppm which can lead to the deactivation of the platinum electrocatalyst in fuel cells.^6^ Among various existing processes for hydrogen purification, the water–gas shift reactions (WGS), the preferential oxidation (PROX), membrane separation and pressure swing adsorption (PSA) routes are the most studied.^7−9^

The water–gas shift (WGS) reaction, CO + H_2_O ↔ CO_2_ + H_2_, is used industrially for hydrogen production to use in ammonia synthesis and in adjusting the CO/H_2_ ratio for further methanol synthesis.^10^ Currently, interest in this reaction is due to its potential use after reforming or gasification processes. Common process products include CO and H_2_. The WGS reaction reduces the CO concentration and converts the additional H_2_, making it very important for fuel cell power generation.^11^

Industrially, the water–gas shift (WGS) reaction is conducted in two adiabatic stages, with each employing a specific catalyst. The first stage, known as the high-temperature shift (HTS), operates in the range of 320–450 °C, while the second stage, the low-temperature shift (LTS), occurs in the range of 200–250 °C. In the HTS stage, catalysts typically consist of iron oxide (Fe_3_O_4_) combined with chromium, which prevents catalyst sintering. For the LTS reaction, CuO-ZnO-Al_2_O_3_ catalysts are employed. However, these catalysts present significant disadvantages, such as their pyrophoric nature, susceptibility to poisoning, and the necessity for extensive pretreatment to avoid deactivation.^10^ As a result, developing an effective catalytic system capable of performing the WGS reaction in a single stage without these limitations has become a central focus of recent studies.

The WGS reaction is exothermic and thermodynamically limited at high temperatures (HT). At low temperatures (LT), the reaction suffers from slow kinetics due to the weak oxidizing nature of H_2_O. Conversely, a medium-temperature shift (MTS) demonstrates faster kinetics, achieving high CO conversion while maintaining equilibrium. Efficient and stable catalytic interfaces within the MTS range are critical to replacing the traditional HT-LT cascade process. Nickel-based catalysts, which typically operate between 300 and 400 °C, have shown optimal performance under these conditions.^12^

Ceria has been extensively studied due to properties that make it a unique material for applications in heterogeneous catalysis: (i) it has excellent redox properties; (ii) it has high oxygen storage capacity; (iii) it shows marked structure-sensitive properties, which can be controlled through synthesis; (iv) it shows both acid–base chemistry; (v) it presents oxygen transport properties; (vi) it is stable under harsh conditions; (vii) its chemical properties are relatively easy to be modified through doping; and (viii) it presents interesting hydrophobic features.^9−11^

The physicochemical and structural properties that govern the catalytic activity of ceria-based materials can be improved by doping with metals. New techniques of electrocatalysis will enable high-precision tunable surface chemistry, allowing for greater control over composition and material interactions. Future synthesis of materials, including single atom doped catalysts with extremely weak oxygen vacancies, as well as decreased energy of dissociating water molecules will allow for extremely efficient hydrogen production through the WGS reaction.^13^

Copper oxide doping of ceria promotes excellent activity in the WGS reaction. The catalytic activity is accentuated due to the strong interactions between the CuO and CeO_2_ phases, influencing the appearance of defects (oxygen vacancies) due to the ease of interaction between the redox pairs Cu^2+^↔ Cu^1+^ and Ce^4+^ ↔ Ce^3+^.^14^ In addition, ceria and copper have lower costs compared with metals used in commercial catalysts, making them competitive. However, when the temperature is higher than 300 °C, the copper particles in the copper-based catalyst are easy to sinter.^15^

One of the ways of preventing the sintering is by adding dopants; among the possible dopants, rare earth elements are of interest because they can easily be incorporated into the cerium lattice. Lanthanum (La) also has proved to be interesting dopants for ceria-based materials: indeed, these elements can enhance the redox properties of ceria and are capable of promoting the generation and effective transfer of highly reactive oxygen species.^11^

She et al.^3^ studied CuO/CeO_2_ catalysts doped with rare earth oxides (RE_2_O_3_, RE = Y, La, Nd, and Sm), prepared by the coprecipitation method. The results revealed that the addition of La_2_O_3_ and Nd_2_O_3_ increased the catalyst activity and stability while adding Y_2_O_3_ and Sm_2_O_3_ the effect was the opposite. The performance of CuO/CeO_2_–Re_2_O_3_ catalysts has been attributed to the dispersion of copper on the surface and the number of oxygen vacancies generated in the ceria lattice. Furthermore, evidence suggests that the interaction of copper oxide with oxygen vacancies on the ceria surface is responsible for the most active sites in the WGS reaction in CuO/CeO_2_ catalysts.

Wang et al.^16^ synthesized a ceria/zirconium catalyst and doped it with rare earth metals like lanthanum (La), neodymium (Nd), praseodymium (Pr), samarium (Sm), and yttrium (Y) and reported that all the metals have increased their activity and selectivity with La, Nd, and Pr performing the best. Rare-earth metals, in general, are known to possess excellent catalytic properties, and when added to a ceria catalyst, they lead to improved thermal stability and enhanced catalytic activity.

Jiang et al.^17^ reported that Cu/Ce/Zr catalysts doped with yttrium (Y) and lanthanum (La), exhibited an increase in oxygen storage capacity (OSC) over pure ceria. The incorporation of ZrO_2_ and La_2_O_3_ in CuO/CeO_2_-based catalysts may improve their activity and stability. This is due to the increased CeO_2_ oxygen storage capacity and reducibility.^10^ Furthermore, the La_2_O_3_ addition in CuO/CeO_2_ hampers catalyst sintering. Thus, it is interesting to study the effect of La on the CuO/CeO_2_ catalysts in the WGS reaction.

Hla et al.^18^ synthesized a CeO_2_–La_2_O_3_-based Cu catalyst via the urea gelation-*co*-precipitation method and studied its kinetics for the WGS reaction at two different reaction temperatures: 550 and 600 °C. This temperature range is well beyond the maximum operating temperature of commercially available catalysts. To demonstrate the CeO_2_–La_2_O_3_-based Cu application under practical reaction conditions, its performance was tested with a wide range of steam-to-carbon ratios in an integral reactor operating mode at an inlet pressure of 20 bar. This catalyst is shown to work well at relatively low steam-to-carbon gas ratios which could provide some practical benefits for a full-scale commercial process.

In the present work, ceria-based materials containing varying amounts of CuO and La_2_O_3_ were prepared via hydrothermal synthesis and evaluated as catalysts for the temperature water–gas shift (WGS) reaction at both low and high temperatures. CuO/La_2_O_3_–CeO_2_ catalysts have been deeply investigated with various characterization techniques and compared with CuO/CeO_2_ catalysts to study the effects of La_2_O_3_ doping on the structure, morphology, defects, and chemical/redox properties. The influence of these characteristics on catalytic activity for the WGS reaction has been discussed. In detail, WGS tests have been carried out under different conditions to evaluate the performance of CuO/CeO_2_ and CuO/La_2_O_3_–CeO_2_ systems in the water–gas shift reaction, assessing their catalytic activity for hydrogen purification and additional H_2_ production.

## 2 Experimental Section

### 2.1 Preparation of Supports and Catalysts

The CeO_2_ and CeO_2_–La_2_O_3_ supports were prepared via a hydrothermal method. Initially, 1.5 g of Ce(NO_3_)_3_·6H_2_O or an equivalent mixture of Ce(NO_3_)_3_·6H_2_O and La(NO_3_)_*x*_·H_2_O (according to the desired CeO_2_–La_2_O_3_ molar ratios of 1:1, 1:2, and 2:1) were dissolved in 30 mL of deionized water under stirring for 15 min. Subsequently, 20 mL of a 10% NaOH solution was added in a single step, and the resulting solution was stirred for an additional 15 min. The mixture was transferred to a 70 mL autoclave and filled to 80% of its total volume with deionized water. The autoclave was heated at 100 °C for 15 h and then cooled to room temperature. The final product was collected, washed thoroughly with deionized water, and calcined under a synthetic air flow (100 mL·min^–1^) at a heating rate of 5 °C·min^–1^ up to 550 °C, where it was maintained for 5 h.

The active phase was introduced into the supports via wet impregnation. An appropriate amount of Cu(NO_3_)_2_·2.5H_2_O was weighed to achieve 8 wt % copper. The copper precursor was dissolved in 30 mL of deionized water and stirred for 15 min. This solution was then impregnated into the precalcined supports using a rotary evaporator at 70 °C, ensuring homogeneous distribution. Following impregnation, the samples were oven-dried for 24 h and subsequently calcined under a synthetic air flow (100 mL·min^–1^ at a heating rate of 2 °C·min^–1^, reaching a final temperature of 400 °C, maintained for 2 h.

All reagents used for the synthesis of supports and catalysts were analytical grade and were supplied by Sigma-Aldrich.

### 2.2 Characterization

#### 2.2.1 Energy-Dispersive X-ray Fluorescence Spectroscopy (ED-XRF)

The chemical compositions of the catalysts were determined using energy-dispersive X-ray fluorescence (ED-XRF) analysis performed on a Shimadzu EDX-720 instrument. It is important to note that the oxygen content could not be determined in this analysis.

#### 2.2.2 Specific Surface Area (*S*_BET_)

The textural properties of the samples were evaluated at −196 °C using nitrogen adsorption–desorption isotherms on a Micromeritics ASAP 2010 instrument. Before the measurements, the samples were pretreated at 200 °C for 2 h under a pressure of 0.1 mbar to remove physisorbed water. The specific surface area was calculated using the Brunauer–Emmett–Teller (BET) method.^19^

#### 2.2.3 X-ray Diffraction (XRD)

The X-ray diffraction (XRD) patterns of the calcined solids were recorded by using a Rigaku Multiflex diffractometer. Scans were conducted over a 2θ range of 5° to 80° at a rate of 2°·min^–1^, employing Cu Kα radiation with a nickel filter. The diffraction patterns were identified by comparison with reference structures in the Joint Committee on Powder Diffraction Standards (JCPDS) database.

#### 2.2.4 Temperature-Programmed Reduction (TPR)

Temperature-programmed reduction (TPR) analyses were performed using a Micromeritics Pulse ChemiSorb 2705 apparatus equipped with a thermal conductivity detector (TCD). For each analysis, 0.03 g of the sample was heated from 25 to 1000 °C under a flow of 10% H_2_/N_2_ (30 mL·min^–1^).

#### 2.2.5 N_2_O Passivation (s-TPR)

The metallic surface area and dispersion of the samples were determined by using the N_2_O passivation method. The same equipment used for the H_2_-TPR measurements was employed for this analysis. It was assumed that no reaction or adsorption of N_2_O occurred on CeO_2_. For the measurements, 0.03 g of the sample was treated under an N_2_ flow (30 mL·min^–1^) at 100 °C for 1 h. After cooling to ambient temperature, the first reduction step (TPR1) was carried out with a 10% H_2_/N_2_ mixture under a flow of 30 mL·min^–1^, using a heating ramp of 10 °C·min^–1^ up to 450 °C, followed by cooling under helium. The sample was then exposed to a flow of N_2_O (30 mL·min^–1^) at 60 °C for 1 h. Finally, a second reduction step (TPR2) was performed with a 5% H_2_/N_2_ mixture under a flow of 30 mL·min^–1^, using a heating ramp of 10 °C·min^–1^ up to 600 °C. The hydrogen consumption ratio before and after oxidation with nitrous oxide was used to calculate copper dispersion. The metallic copper area of the samples was determined based on the number of surface atoms per unit area, using a value of 1.47 × 10^19^ Cu atoms/m^2^.^6^

#### 2.2.6 Oxygen Storage Capacity (OSC)

Oxygen storage capacity (OSC) measurements were conducted by using a quartz reactor in a heating line coupled with a Pfeiffer QMS200 quadrupole mass spectrometer. The samples were heated to 350 °C at a heating rate of 10 °C·min^–1^ under a continuous helium flow of 50 mL·min^–1^ at atmospheric pressure. Once the target temperature was reached, a 10% O_2_/He mixture (corresponding to 4 mL·min^–1^ O_2_) was introduced for 10 min to fully oxidize the sample. Following oxidation, helium was used at a flow rate of 50 mL·min^–1^ to remove excess weakly adsorbed O_2_ from the sample. Subsequently, 10 pulses of a 10% CO/N_2_ mixture (8 mL·min^–1^ CO per pulse) were injected into the reactor, each lasting 3 min. The CO_2_ produced from the reaction between injected CO and oxygen incorporated into the solid was quantified. After each pulse, helium was introduced into the system for 17 min to ensure the complete removal of CO_2_ before the next pulse. The OSC was determined based on the CO_2_ produced during the first CO pulse. The total oxygen storage capacity (OSCC) was calculated as the total amount of reactive oxygen, represented by the sum of CO converted to CO_2_ across all 10 pulses.

#### 2.2.7 In Situ X-ray Diffraction (In Situ XRD)

In situ X-ray diffraction (XRD) measurements were performed in reflection mode over a 2θ range of 35° to 70°, using Cu-Kα radiation with an energy resolution of 4.3 eV. The instrument was calibrated with a Si (111) monochromator. The oxidized samples were heated in situ under an H_2_ atmosphere from 25 to 300 °C at a heating rate of 10 °C·min^–1^ and maintained at 300 °C for 30 min. Subsequently, the temperature was increased to 350 °C under a N_2_ atmosphere. The reaction was conducted using a CO:H_2_ molar feed ratio of 1:5 at 350 °C, employing the same real and ideal reaction flow conditions used in the catalytic tests. The apparent crystallite sizes of the catalysts were determined using Scherrer’s equation.^20^

#### 2.2.8 X-ray Absorption Near Edge Structure (XANES)

Absorption measurements were performed at the Cu K-edge (8979 eV) and the Ce LIII-edge (5724 eV). In situ XANES measurements were carried out during H_2_-TPR under a 5% H_2_/He flow, starting from room temperature to 300 °C at a flow rate of 30 mL·min^–1^, with a heating rate of 10 °C·min^–1^. The system was maintained at these conditions for 30 min. Subsequently, the temperature was increased to 350 °C, and the reaction mixture was introduced to initiate the reaction. The catalysts were exposed to a CO:H_2_ mixture with a molar ratio of 1:5. The samples were prepared in the form of pellets (100 mg total mass), consisting of 30 mg of sample diluted in boron nitride, and placed in a quartz tubular furnace with sealed KAPTON-refrigerated windows for transmission mode measurements. The energy edge positions of the reference spectra for CeO_2_ and CeOHCO_3_, reflecting the ceria oxidation state, were determined by using a combinatorial linear fitting model. X-ray absorption spectroscopy (XAS) data were processed using Athena software.

### 2.3 Catalytic Tests

The catalysts were tested in both ideal and real WGS reactions. These reactions differ in the gas mixture composition of the reactor feed. In the ideal WGS reaction, only CO and H_2_O vapors were used. In the real WGS reaction, H_2_ and CO_2_ were added to CO and H_2_O to more closely simulate the conditions of the industrial process.

The WGS reactions were carried out in a quartz reactor, using 180 mg of catalyst for the ideal WGS reaction and 311 mg for the real WGS reaction, maintaining the same catalyst weight-to-reactant flow ratio for future comparison. All catalysts were reduced under a flow of 30 mL/min of pure hydrogen, with the temperature ramped at a rate of 10 °C·min^–1^ until reaching 300 °C, where it was maintained for 1 h.

For the ideal WGS reaction, the temperature was varied in steps with 50 °C intervals, with the initial and final temperatures set to 150 and 350 °C, respectively. Four injections were made at each temperature after sufficient time was allowed for the reaction system to stabilize. The two most active catalysts at 350 °C in the ideal WGS reaction underwent 24 h catalytic stability tests under both ideal and real WGS reaction conditions.

The real WGS reaction was performed with a CO/N_2_ feed at a flow rate of 5.0 mL·min^–1^, at 25 °C and 1.0 atm, corresponding to 2.0 × 10^–4^ mol·min^–1^ of CO. The CO/N_2_ gas mixture contained 10% CO by molar ratio. The CO:H_2_O molar ratio was set to 1:5 to favor the real WGS reaction. The flow rates were controlled using a mass flow controller, with the controller set-points maintained constant throughout all catalytic tests. The gases, supplied by White Martins, had a purity of 99.99% to ensure strict control over the catalytic tests.

The composition of the reactor effluent was determined by gas chromatography using a VARIAN 3800 chromatograph equipped with two thermal conductivity detectors (TCDs), with helium and nitrogen as carrier gases. Gas flow rates were controlled by a mass flow controller (MKS Instruments, model 247, with four channels). Liquid water was introduced into the system through a high-pressure pump with a digital flow control. Water vapor was generated by a vaporizer maintained at 300 °C.

CO conversion was calculated based on the carbon balance in the reactor, in which all carbon introduced into the system as carbon monoxide (CO^o^) was converted either to carbon dioxide (CO_2_) or remained as unreacted carbon monoxide (CO^S^).

## 3 Results and Discussion

Table 1 presents the mass values of Cu, Ce, and La elements in the calcined samples, as determined by ED-XRF. The experimental mass results are consistent with the nominal mass values.

**Table 1:** Nominal and Experimental Mass Values of the Elements Present in the Samples

sample	nominal (% m/m)			experimental (% m/m)		
	Cu	Ce	La	Cu	Ce	La
CuO/CeO_2_	9.8	90.2		11.5	88.5	
CuO/2CeO_2_-1La_2_O_3_	9.6	45.4	45.0	10.9	47.9	41.3
CuO/1CeO_2_-1La_2_O_3_	9.5	30.2	60.3	10.2	33.0	56.2
CuO/1CeO_2_-2La_2_O_3_	9.5	18.2	72.3	9.8	24.1	66.1

**Figure 1:**
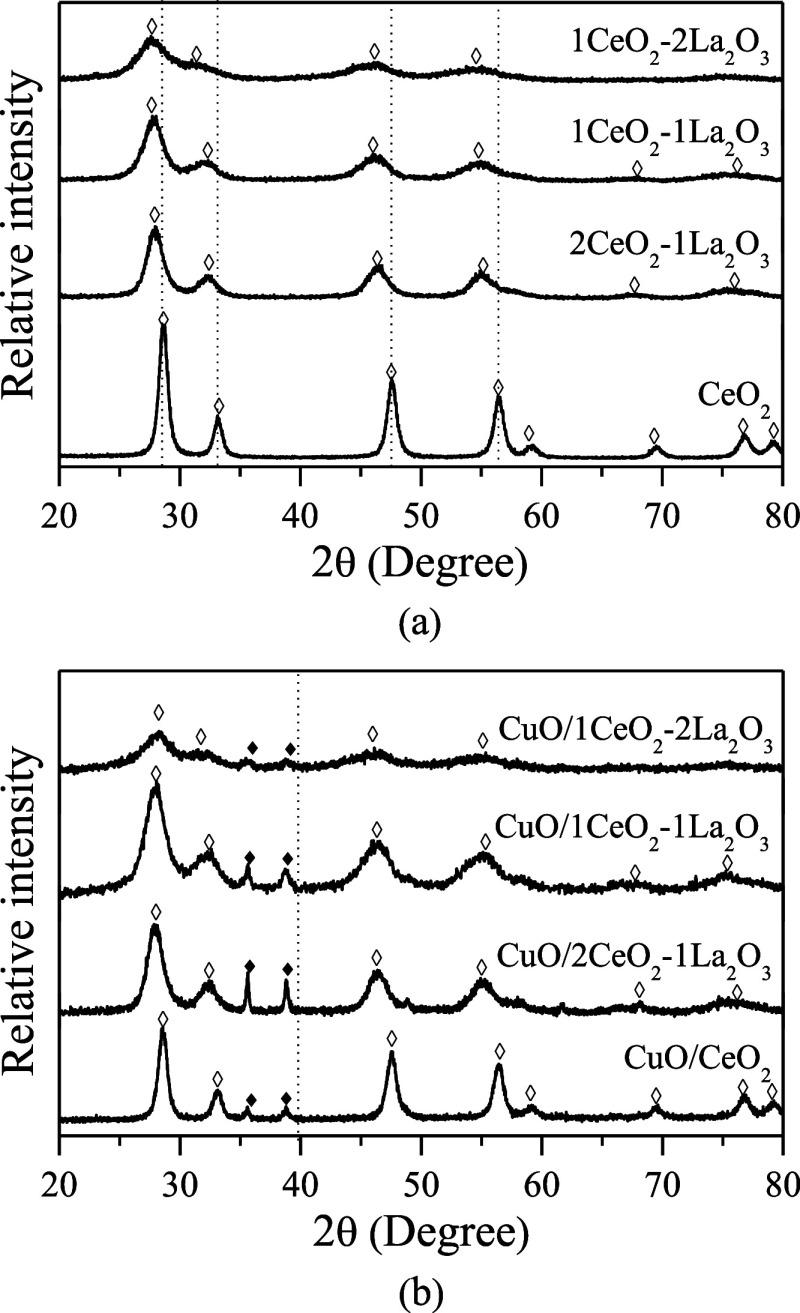
X-ray diffraction patterns: (a) supports and (b) supported catalysts (◊ CeO_2_ and ◆ CuO).

Figure 1a,b shows the supports and calcined catalysts XRD patterns, respectively. Only peaks related to the CeO_2_ phase of the cubic fluorite type (2θ = 28.6°, 33.2°, 47.5°, 56.4°, 59.2°, 69.5°, 76.9°, and 79.3°) are observed in both supports and lanthanum-containing catalysts. The absence of nonstoichiometric lanthanum species (LaO_*x*_, La(OH)_3_, La_2_O_2_CO_3_, etc.) confirms that there is no phase segregation in these samples. On the other hand, an apparent shift of the peaks to smaller 2θ angles can be seen in the CeO_2_ diffractogram, as well as a decrease in intensity and a broadening of these peaks. This displacement in CeO_2_ diffraction peaks indicates the formation of solid solutions of the LaCeO_*x*_ type, and the observed decrease in diffraction peak intensity may be due to the high absorption of X-rays by the lanthanum atoms.^22−24^ Additionally, the incorporation of La^3+^ into the CeO_2_ lattice should increase the lattice parameter value, since the ionic radius of La^3+^ (0.106 nm) is greater than that of Ce^4+^ (0.094 nm).^22,23^

In Figure 1b, it is also observed that as the La content increases, the material becomes less crystalline and only the most intense peaks of the CeO_2_ structure are noticeable. For these catalysts, it can be noted that all have diffraction peaks referring to the CuO compound (2θ = 35.6° and 38.8°), besides the peaks related to fluorite-type CeO_2_. Samples containing lanthanum continue to present diffraction peak displacement concerning CeO_2_.

Table 2 presents the crystallite sizes of copper (*D*_CuO_) and cerium (*D*_CeO_2__), calculated using Scherrer’s equation based on the diffraction peaks at 2θ = 28.6° (CeO_2_) and 2θ = 35.6° (CuO), as shown in the diffractograms in Figure 1. The results indicate that the additives inhibit the growth of CeO_2_ crystallites, promoting smaller particles, possibly due to the formation of a less crystalline structure with broader peaks in the presence of lanthanum.^21^ When the CeO_2_ molar ratio increases, the apparent ceria crystallite size also increases. This effect is attributed to the formation of a solid solution between La_2_O_3_ and CeO_2_, which retards crystallite growth and forms a more thermodynamically stable phase.^14^ Furthermore, it is observed that the average CuO crystallite size increases with the addition of La_2_O_3_ in the CuO/2CeO_2_-1La_2_O_3_ and CuO/1CeO_2_-1La_2_O_3_ samples, indicating less dispersion of these particles on the catalyst surfaces. For the CuO/2 CeO_2_-1La_2_O_3_ sample, the CuO crystallite size is larger due to the higher amount of ceria present, which likely inhibited the growth of CuO crystals. On the other hand, the CuO/1 CeO_2_-1La_2_O_3_ sample exhibits the largest copper crystallite size, possibly due to the formation of a solid solution between lanthanum and ceria, as evidenced by the XRD analyses.

**Table 2:** Experimental Specific Surface Area (*S*_BET_) and Crystallite Size (*D*) Values

sample	*S*_BET_ (m^2^·g_cat_^–1^)		*D*_CeO_2__ (nm)		*D*_CuO_ (nm)
	support	catalyst	support	catalyst	catalyst
CuO/CeO_2_	107	90	9.4	6.6	9.9
CuO/2CeO_2_-1La_2_O_3_	90	86	5.0	4.8	13.9
CuO/1CeO_2_-1La_2_O_3_	78	57	3.9	3.9	10.3
CuO/1CeO_2_-2La_2_O_3_	80	23	2.7	2.8	7.0

Table 2 also shows the BET-specific surface area (*S*_BET_) before the reactions. From the surface area results, obtained through N_2_ physisorption analysis for the supports and catalysts calcined, it has been noted that adding La_2_O_3_ to the samples containing ceria generally results in lower specific surface area values compared to the pure ceria samples.^25^ It has also been observed that impregnating CuO on the supports leads to a decrease in the surface area of the samples. This decrease may be due to the coating of support pores by CuO and the inherently low surface area of CuO, resulting in the catalyst surface area decrease.^26^ By a comparison of the samples, it is observed that there are slight changes in the specific surface area with the addition of CuO, except for the CuO/1CeO_2_-2La_2_O_3_ catalyst, which shows a significant decrease (58 m^2^·g^–1^).

**Figure 2:**
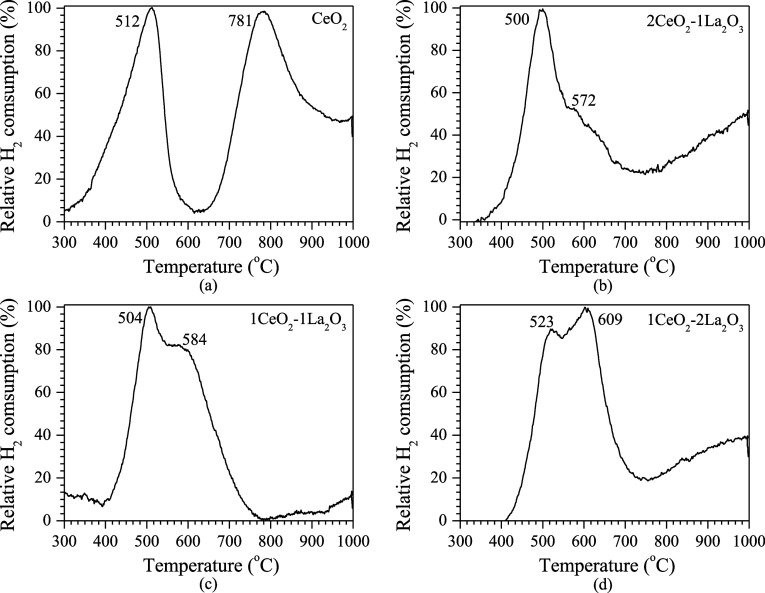
TPR supports the patterns.

Figure 2 shows the reduction profiles of the supports calcined at 550 °C. It can be observed that only the pure ceria sample shows a peak at 781 °C (Figure 2a), which is attributed to the bulk reduction of Ce^4+^ to Ce^3+^.^27,28^ The peak in the 500–519 °C range corresponds to the reduction of surface ceria (Ce^4+^ to Ce^3+^).^23^ For the catalysts containing La_2_O_3_, the peak observed around 550–600 °C is attributed to the reduction of carbonate and/or hydroxyl species formed due to the removal of H_2_O and CO_2_ from the La compounds (La_2_O_2_CO_3_, La(OH)_3_, etc.). As the ceria content increases in the sample, the reduction of these species occurs at lower temperatures. This enhanced reduction is likely due to the strong interaction between La and Ce species.^23,29,30^ The hydrogen consumption analysis presented in Table 3 supports this observation.

**Table 3:** Quantitative Analysis of Hydrogen Consumption by the Calcined Support

samples	total H_2_ consumption (μmol.g_cat_^–1^)
CeO_2_	276
2CeO_2_-1La_2_O_3_	264
1CeO_2_-1La_2_O_3_	337
1CeO_2_-2La_2_O_3_	200

Table 3 shows the quantitative results of total H_2_ consumption by the calcined support during the reduction process. The peak at 609 °C has been not counted in the total value of H_2_ consumption, as these are related to carbonate and/or hydroxide compounds.^23,28,31^ Some researchers have reported that La_2_O_3_-doped ceria exhibits a reduction profile similar to pure ceria but with surface peaks slightly shifted to lower temperatures. Additionally, they found that hydrogen consumption increases significantly with higher concentrations of lanthanum.^28,29^ However, Zhang et al.^23^ observed that for La_2_O_3_ contents greater than 60%, Ce^4+^ species are surrounded by La^3+^ species which, as they are more electronegative, promote the electrons transfer “La ← O″, shifting the reduction peaks CeO_2-x_ nonstoichiometric for higher temperatures. As a result, the intensity of nonstoichiometric CeO_2-x_ peak reduction decreases significantly and the fluorite-like structure is gradually transformed into a typical La_2_O_3_ structure. The results of total H_2_ consumption shown in Table 3 follow the pattern observed by these researchers; that is, there is an optimal lanthanum content that favors the support reduction.

**Figure 3:**
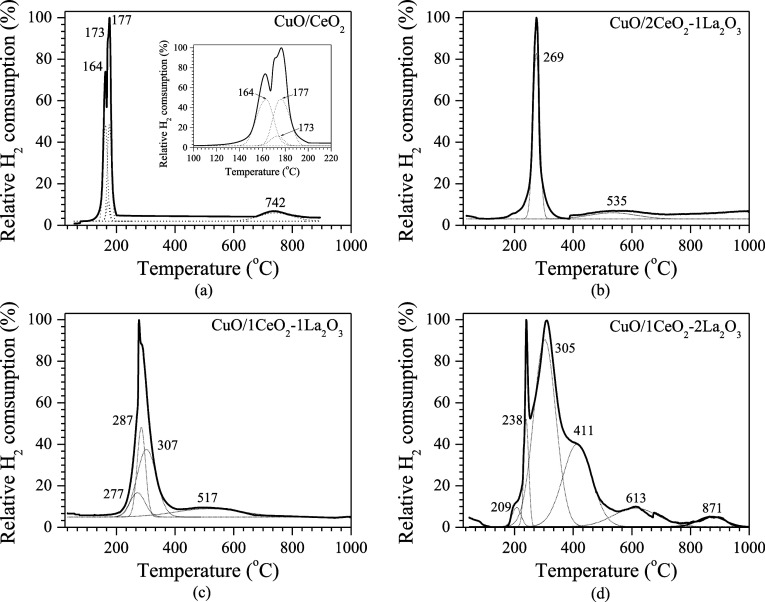
Catalysts TPR patterns: (a) CuO/CeO_2_; (b) CuO/2CeO_2_-1La_2_O_3_; (c) CuO/1CeO_2_-1La_2_O_3_; and (d) CuO/1CeO_2_-2La_2_O_3_.

Figure 3 shows the reduction profiles of CuO-modified supports calcined at 400 °C. Deconvolution has been applied to the H_2_-TPR spectra for all samples. It is observed that the addition of La_2_O_3_ shifts all reduction peaks to lower temperatures compared to CuO/CeO_2_. This result suggests that La_2_O_3_ increases reducibility, probably due to structural changes induced in the ceria lattice when Ce^4+^ cations are replaced by La^3+^ cations, favoring the diffusion of O^2–^ anions into the lattice.^3,32^

In all spectra in Figure 3, the CuO reduction peak is also observed at temperatures below 300 °C. The decomposition of the CuO reduction peak into three peaks can be visualized in the catalysts CuO/CeO_2_, CuO/1CeO_2_-1La_2_O_3_, and CuO/1CeO_2_-2La_2_O_3_. The first two peaks are attributed to the reduction of noncrystalline CuO in close contact with cerium and crystalline CuO interacting with oxygen vacancies on the cerium surface, respectively. The third peak is attributed to the surface cerium reduction, the CuO incorporated into the cerium lattice, and the large isolated phase of crystalline CuO, which is not associated with CeO_2_.^3^ By increasing the La_2_O_3_ content in the catalyst, an increase in the species reduction temperature occurs, and thus a higher hydrogen consumption is noted. As observed in Figure 3d, the CuO/1CeO_2_-2La_2_O_3_ catalyst shows the highest number of reduction peaks (6 peaks), indicating that this catalyst exhibits different interactions between CuO and the support compared with the other catalysts. The peaks above 414 °C up to 535 °C can be attributed to the CeO_2_ and/or La_2_O_3_ reduction which is strongly bound.^30^ The peaks below 284 °C are related to the CuO surface species reduction. The peaks at intermediate temperatures are related to the reduction of CuO species present in the bulk, which have larger particles. The peak at 613 °C, present only in the CuO/1CeO_2_-2La_2_O_3_ catalyst, suggests the H_2_O and/or CO_2_ removal from La compounds.^25^

Table 4 shows the dispersion and metallic area results. The metallic area calculations have been performed assuming a density of 1.46 × 10^19^ atoms Cu/m^2^ as reported in the literature.^3,33^ The presence of La_2_O_3_ in the CuO/CeO_2_ samples causes a decrease in the metallic area and its dispersion too. This result is consistent with H_2_-TPR (Figure 3) and with the specific surface area values shown in Table 2. The CuO/CeO_2_ catalyst exhibits higher metallic area and dispersion values than those of the catalysts doped with La_2_O_3_. As previously discussed, the addition of La_2_O_3_ may reduce particle size and increase catalytic activity and stability, but metallic dispersion is not favored due to the lower dispersion of La_2_O_3_ at high La content.

**Table 4:** Catalysts Metallic Area and Dispersion

sample	metallic area (m^2^·g_cat_^–1^)^a^	dispersion (%)
CuO/CeO_2_	61.0	31.5
CuO/1CeO_2_-1La_2_O_3_	19.5	10.0
CuO/2CeO_2_-1La_2_O_3_	17.0	8.8
CuO/1CeO_2_-2La_2_O_3_	16.2	8.3

a g_cat_ = catalyst gram.

**Figure 4:**
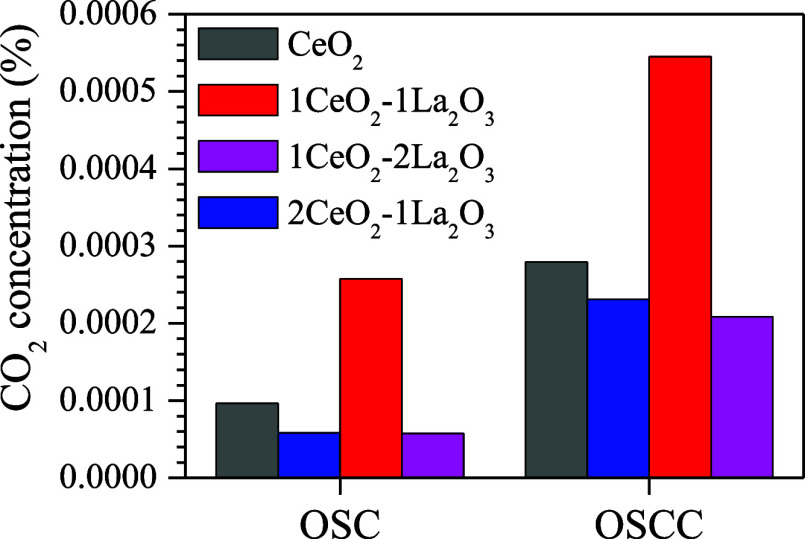
Oxygen storage capacity (OSC) and oxygen storage capacity complete (OSCC).

Figure 4 shows the oxygen storage capacity (OSC) for the first CO pulse and after 10 CO pulses (OSCC). Cerium oxide is known for its high oxygen exchange capacity, a property typical of ceria due to the fast reversibility of the Ce^3+^/Ce^4+^ redox cycle.^34,35^ When two La^3+^ ions replace two Ce^4+^ ions in ceria, an oxygen vacancy is created, which enhances the oxygen diffusion rate and, consequently, the OSC.^34,36^ Catalysts containing La_2_O_3_ have shown the highest OSC, correlating with increased CO_2_ production during both the OSC and the OSCC. This suggests that La induces significant changes in the chemical properties of the catalysts, particularly through the generation of oxygen vacancies. The extent of this effect is influenced by the La content in the catalysts.^3,34^

**Figure 5:**
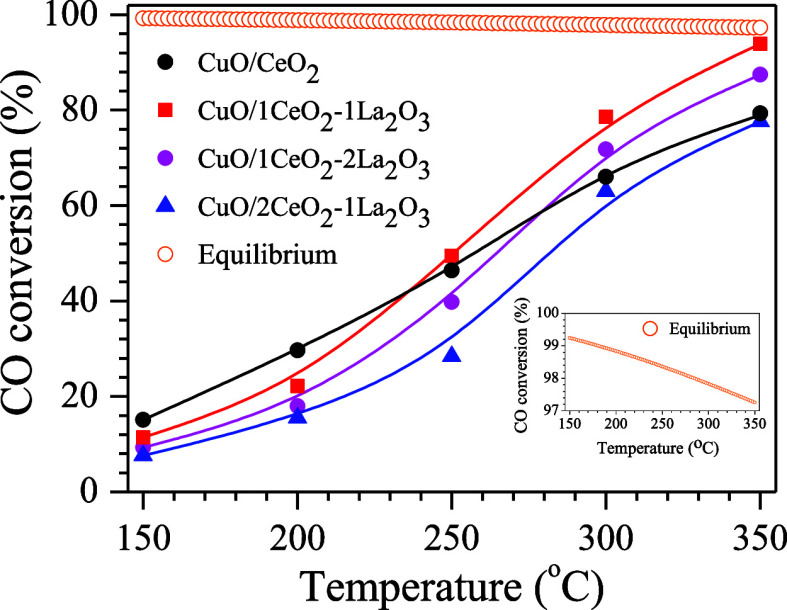
CO conversion as a function of temperature for the ideal WGS reaction. (CO flow rate = 5 mL·min^–1^; CO:H_2_O molar ratio = 1:5).

Figure 5 shows the CO conversion as a function of temperature for the ideal WGS reaction using calcined catalysts. Previous tests of the calcined supports under the same conditions resulted in CO conversions below 4%. The performance of the catalysts is consistent with the *S*_BET_ and TPR analysis results, indicating that a larger surface area, as well as a larger and more dispersed metallic area, correlates with a higher catalytic activity. Across all temperatures, the CuO/2CeO_2_-1La_2_O_3_ catalyst exhibited lower CO conversions compared with the other catalysts, likely due to its lower specific surface area, smaller metallic area, and reduced metallic dispersion. At temperatures below 250 °C, the CuO/CeO_2_ catalyst outperformed CuO/1CeO_2_-1La_2_O_3_, suggesting that in this temperature range, the benefits of the larger surface area and higher metallic dispersion of CuO/CeO_2_ outweigh the greater oxygen storage capacity of CuO/1CeO_2_-1La_2_O_3_ (Figure 4). In addition, this superior catalytic activity of the CuO/CeO_2_ catalyst at lower temperatures is due to the complete reduction of CuO in this sample under the reaction conditions. However, above 250 °C, the CuO/1CeO_2_-1La_2_O_3_ and CuO/1CeO_2_-2La_2_O_3_ catalysts demonstrated better catalytic activity, likely due to their greater reducibility compared to the CuO/CeO_2_ catalyst.^3^

The equilibrium curve, as shown in Figure 5, indicates that even at high temperatures, the CO conversion remains below 100%. However, at elevated temperatures, the differences among the catalysts become less pronounced, suggesting that increased temperature positively influences the catalytic activity. The WGS reaction is moderately exothermic, and its equilibrium constant decreases with rising temperatures. At lower temperatures, the conversion through the WGS reaction is kinetically limited, while at higher temperatures, chemical equilibrium is quickly achieved.^10,18,37^

**Figure 6:**
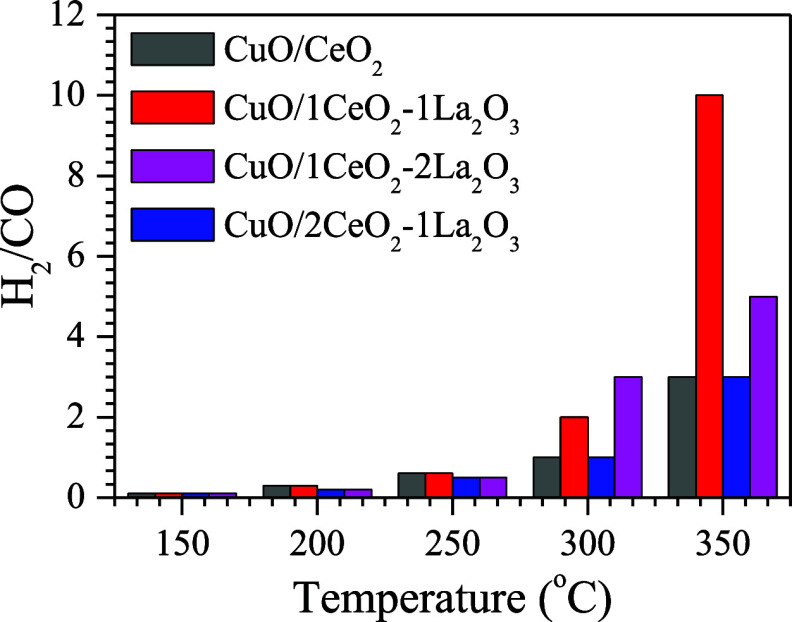
H_2_/CO ratio measured at the reactor outlet.

Figure 6 shows the H_2_/CO ratio produced during the ideal WGS reactions for each catalyst tested at various temperatures. As expected, the H_2_/CO molar ratio decreases with decreasing temperature, corresponding to the lower CO conversion in the ideal WGS reaction under these conditions. The CuO/1CeO_2_-1La_2_O_3_ catalyst exhibited the highest H_2_/CO molar ratio, approximately 10, indicating that this catalyst achieved the highest H_2_ production at 350 °C.

**Figure 7:**
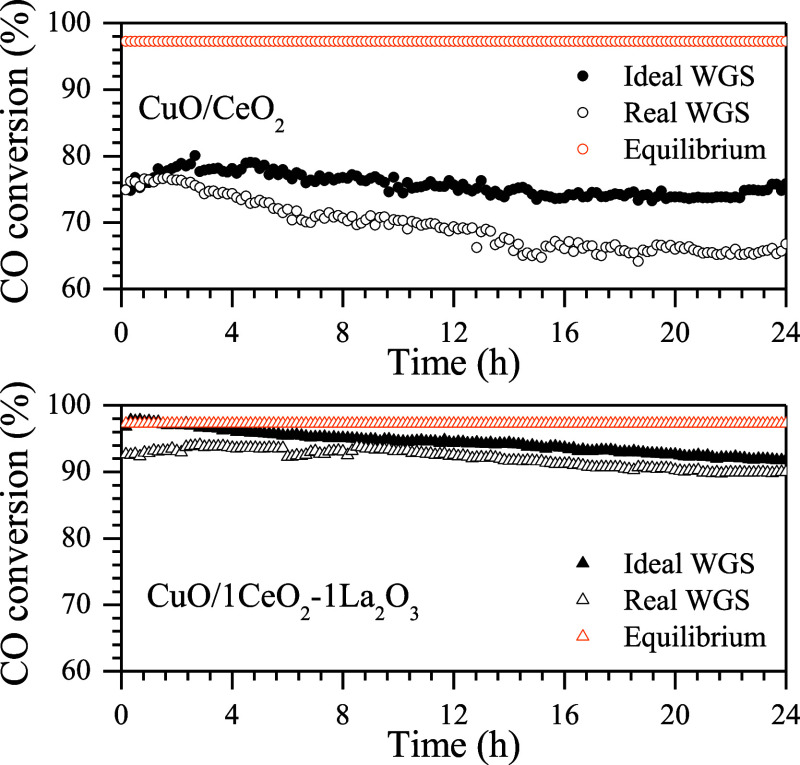
CO conversion as a function of time for the ideal and real WGS reactions carried out at 350 °C using the CuO/CeO_2_ and CuO/1CeO_2_-1La_2_O_3_ catalysts. (CO flow rate = 5 mL·min^–1^; CO:H_2_O molar ratio = 1:5).

Figure 7 presents the stability test results for the ideal and real WGS reactions at 350 °C, using CuO/CeO_2_ and CuO/1CeO_2_-1La_2_O_3_ catalysts. Under both reaction conditions, the CuO/1CeO_2_-1La_2_O_3_ catalyst demonstrated higher activity than that of CuO/1CeO_2_. After 24 h of reaction, the CuO/1CeO_2_ catalyst exhibited a 6% decrease in conversion, whereas the CuO/1CeO_2_-1La_2_O_3_ showed only a 3% decrease. These results suggest that the La_2_O_3_ addition enhances the stability of CuO/CeO_2_-catalysts materials. At temperatures above 300 °C, copper sintering may occur, leading to a loss of metallic surface area and, consequently, a decrease in catalytic activity. However, this effect is less pronounced in the CuO/1CeO_2_-1La_2_O_3_ catalyst due to increased resistance to sintering promoted by La_2_O_3_ doping.^38,39^ The presence of CO_2_ and H_2_ in the feed during the reaction negatively affected the performance of both catalysts; however, the effect was more pronounced in the CuO/CeO_2_ catalyst. This reduction in conversion was expected as the addition of products to the feed shifts the equilibrium of the reversible reaction toward the formation of reactants. Moreover, CO_2_ may induce the formation of carbonate species on the catalyst surface, blocking active sites and promoting sintering, as well as competing with CO for the same sites, thereby reducing reactant conversion.^14^ In summary, the lanthanum-doped catalyst exhibits a greater resistance to activity loss under real WGS reaction conditions. Similar findings were reported by Stephanopoulos and collaborators, whose results show that a catalyst composed of 10 atomic percent copper supported on cerium oxide doped with 30 atomic percent lanthanum rapidly lost some activity at the beginning but stabilized after 20 h on stream.^10,18^

Table 5 shows the catalysts’ specific surface area after being tested in the ideal and real WGS reactions. The first row shows the catalysts’ specific surface area tested at reaction temperatures ranging from 50 to 350 °C. The other two rows display the catalysts’ specific surface area after the stability test, which involves reaction at 350 °C for 24 h. Comparing the specific surface area values before the raw catalysts (Table 2, second column) with specific surface area values after the catalysts were tested in the WGS reaction (Table 5, first column), we noted a decrease in the specific surface area of all catalysts after WGS reactions. The catalyst without La_2_O_3_ showed the greatest decrease in specific surface area compared to that of the La_2_O_3_-doped catalysts. For instance, CuO/CeO_2_ showed a decrease of around 30 m^2^·g_cat_^–1^, while CuO/2CeO_2_-1La_2_O_3_ showed a drop of around 17 m^2^·g_cat_^–1^. The CuO/1CeO^2^-1La_2_O_3_ catalyst showed a smaller decrease in the specific surface area (2.4 m^2^·g_cat_^–1^), indicating that this catalyst experienced the lowest sintering of metallic copper particles. This process is responsible for the loss of catalyst activity due to the reduction in the area of the active sites.

**Table 5:** Catalysts Specific Surface Areas after WGS Reactions

catalyst	S_BET_ (m^2^g_cat_^–1^)		
	50 to 350 °C	24 h at 350 °C	
	ideal WGS	ideal WGS	real WGS
CuO/CeO_2_	60.2	49.8	56.4
CuO/2CeO_2_-1La_2_O_3_	68.5		
CuO/1CeO_2_-1La_2_O_3_	54.6	55.4	55.3
CuO/1CeO_2_-2La_2_O_3_	17.9		

Upon examination of Table 5, columns two and three present the specific surface area values of the CuO/CeO_2_ and CuO/1CeO_2_-1La_2_O_3_ catalysts after testing in both the ideal and real WGS reactions at 350 °C over 24 h. It was observed that the most significant decrease in the surface area occurred in the catalyst without La_2_O_3_ (approximately 40 m^2^·g_cat_^–1^). In contrast, the CuO/1CeO_2_-1La_2_O_3_ catalyst exhibited only a 2.4 m^2^·g_cat_^–1^ decrease in the rate of CO conversion, indicating greater resistance to sintering. This result is supported by the stability reaction tests shown in Figure 7. When CO_2_ and H_2_ were added to the feed stream, the CuO/CeO_2_ catalyst showed a smaller decline in performance in the real WGS compared to that in the ideal WGS. Conversely, the CuO/1CeO_2_-1La_2_O_3_ catalyst displayed no significant change in specific surface area, further demonstrating its resistance to sintering even with the addition of CO_2_ and H_2_ to the feed stream.

**Figure 8:**
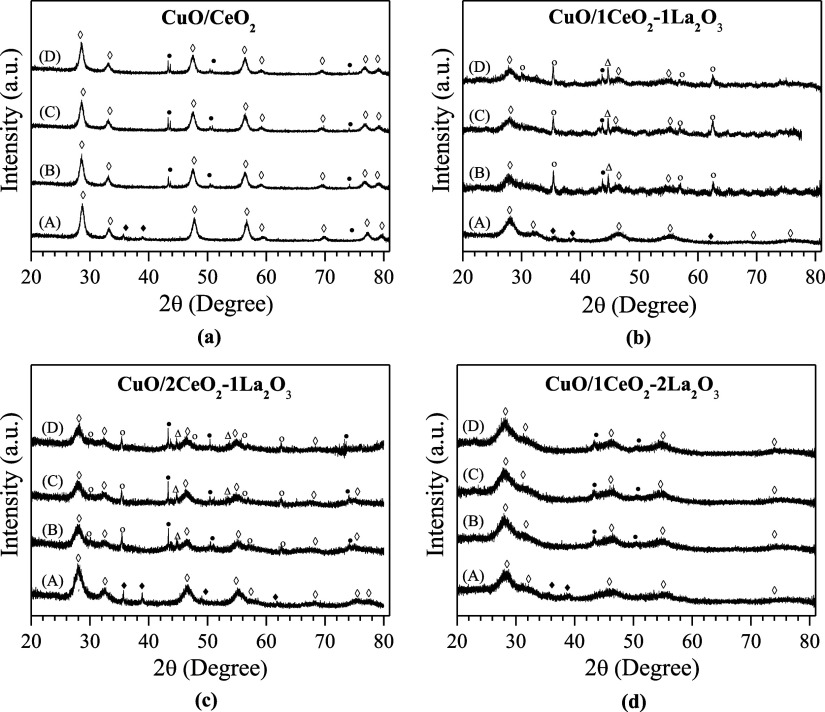
In situ XRD diffractograms for catalysts: (a) CuO/CeO; (b) CuO/1CeO_2_-1La_2_O_3_; (c) CuO/2CeO_2_-1La_2_O_3_; and (d) CuO/1CeO_2_-2La_2_O_3_. (◊ CeO_2_; ◆ CuO; ● Cu^0^; ○ La_2_O_2_CO_3_).

Figure 8 shows the in situ XRD results performed during H_2_-TPR analysis and WGS reactions of the studied catalysts. For each catalyst, one graph is presented containing four XRD diffractograms. Each diffractogram was obtained under a specific condition, as follows: (A) around 25 °C; (B) H_2_-TPR at 300 °C; (C) ideal WGS at 350 °C; and (D) real WGS at 350 °C. In the XRD profiles, the species are represented by one symbol: ◊ CeO_2_; ◆ CuO; ● Cu^0^; and * La_2_O_2_CO_3_. For all catalysts, the cubic fluorite-type CeO_2_ phase (◊) is present in all profiles. These results indicate that ceria is stable under different test conditions. The Ce^3+^ presence during reactions is commonly mentioned in the literature; however, in this work, it has not been possible to identify Ce^3+^ by XRD analysis. The CuO/CeO_2_ catalyst exhibited a more crystalline structure than those containing La, indicating that lanthanum oxide modifies the ceria structure. At 25 °C, all diffractograms showed typical monoclinic tenorite peaks of the CuO phase (◆); one peak at 35.6° and the other at 38.8°. It has not been possible to identify the Cu_2_O phase presence in any catalyst, indicating that, there has been a CuO → Cu^0^ reduction.^3^ In the WGS reaction tests, the in situ XRD analysis has identified the metallic copper in the face-centered-cubic phase (●) in all catalysts. This phase corresponds to the peaks at 43.3° and 50.5° in all of the diffractograms shown in Figure 8. In the catalysts containing La, copper stabilization is possible even under hydrogen-rich feed conditions.^35^ The metallic copper presence in all diffractograms enhances the sintering occurrence during the reduction at 300 °C and also during the real and ideal WGS reactions. The XRD results are consistent with the H_2_-TPR analysis shown in Figure 3. The catalytic tests showed that the higher the reaction temperature, the higher the activity of all catalysts in the presence of metallic copper.^36^

After reduction with pure H_2_, all catalysts showed no characteristic CuO peak, indicating that complete reduction or high dispersion had occurred. The CuO/1CeO_2_-1La_2_O_3_ and CuO/2CeO_2_-1La_2_O_3_ catalysts exhibit a La_2_O_3_ hexagonal phase. In fact, the peaks corresponding to the hexagonal phase appeared only after reduction at 300 °C, suggesting possible structural segregation. It is likely that a La(OH)_3_ structure was formed during preparation. The CuO/1CeO_2_-2La_2_O_3_ catalyst showed no such phases, indicating the absence or high dispersion of these compounds over this catalyst.

Table 6 shows the Cu^0^ crystallite size values observed after activation with H_2_ at 300 °C, ideal WGS, and real WGS conditions by XRD analysis in situ. Note that the two catalysts with the highest La concentrations presented the lowest Cu^0^ crystallite size values, indicating that La favors its dispersion. This result is consistent with the specific surface area after the reaction (Table 3), where these catalysts showed a smaller decrease in the specific surface area after being used in the WGS reaction.

**Table 6:** Average Cu^0^ Crystallite Size Values

sample	*D* (nm)		
	reduction	ideal WGS	real WGS
CuO/CeO_2_	26	39	52
CuO/2CeO_2_-1La_2_O_3_	46	40	56
CuO/1CeO_2_-1La_2_O_3_	19	12	15
CuO/1CeO_2_-2La_2_O_3_	12	11	10

**Figure 9:**
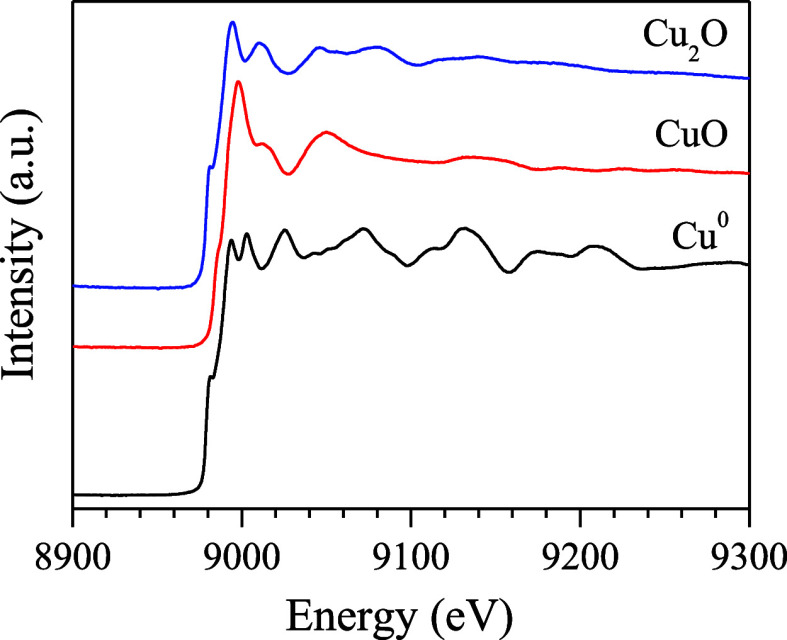
XANES spectra at the Cu K-edge of the Cu standards.

Figure 9 shows the XANES spectra of Cu^0^, Cu_2_O, and CuO at the Cu K-edge, conducted to determine the oxidation states of copper in the catalysts. The spectra were normalized to enhance the identification of pattern differences. The CuO spectrum is characterized by two absorption peaks: one at 8985 eV and the other at 8997 eV. The first peak of weak absorption is assigned to the 1s–3d transition. The second peak of a stronger absorption is due to the allowed Cu^2+^ 1s–4p dipole transitions, which occur in this absorption band and are generally called the white line. On the other hand, the Cu^0^ XANES spectra exhibit an absorption band at 8981.8 eV, with a well-defined doublet in the postedge region. The absorption edge for Cu_2_O, due to the 1s → 4p_*xy*_ electronic transition, appears at 8982 eV. The reference values for the absorption band patterns are consistent with literature reports.^40−42^

**Figure 10:**
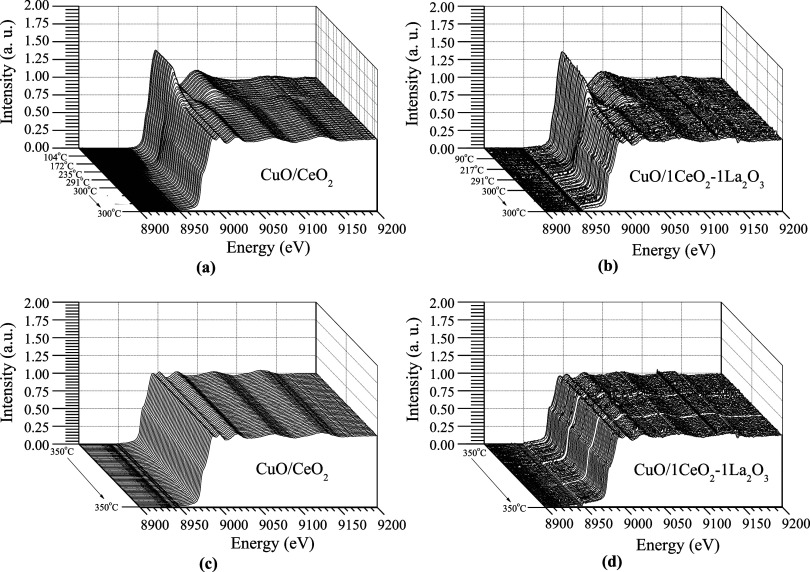
In situ XANES spectra performed at the Cu edge for the CuO/CeO_2_ and CuO/1CeO_2_-1La_2_O_3_ catalysts: (a, b) reduction process with H_2_ at 300 °C; (c, d) ideal WGS reaction at 350 °C.

Figure 10a,b shows the in situ XANES spectra performed at the Cu edge during the reduction process for the CuO/CeO_2_ and CuO/1CeO_2_-1La_2_O_3_ catalysts, respectively. These catalysts were reduced from room temperature to 300 °C and then kept at that temperature for 30 min under a hydrogen atmosphere. For both catalysts, the spectra at room temperature are similar to that of CuO (Figure 9), showing an intense absorption peak at 8997 eV. As the temperature increases, the intensity of the white line decreases, causing the spectra to gradually resemble the Cu^0^ profile (Figure 9). This change in profile is attributed to the reduction of Cu^2+^ to Cu^0^. The transition in the oxidation state begins at 180 and 240 °C for the CuO/CeO_2_ and CuO/1CeO_2_-1La_2_O_3_ catalysts, respectively. As previously mentioned, the La_2_O_3_ addition to the CuO/CeO_2_ catalyst has promoted a strong interaction between CuO and the support, hindering its reduction. These results are consistent with those obtained in the H_2_-TPR analysis (Figure 3).

After the reduction process, the furnace temperature was raised to 350 °C, and then the reactor was fed with the reactants under the same conditions as the ideal WGS reaction. Figure 10c,d shows the in situ XANES spectra performed at the Cu edge during the real WGS reaction for the CuO/CeO_2_ and CuO/1CeO_2_-1La_2_O_3_ catalysts, respectively. For both catalysts, a shift of the absorption band to lower energies was observed throughout the reduction and reaction processes. By the end of the reaction, the spectra continued to exhibit Cu^0^ characteristics. This suggests that in these catalysts, the copper species are reduced in a single step (Cu^2+^ → Cu^0^), without the formation of an intermediate Cu^1+^ state. These results are also in accordance with the in situ XRD and H_2_-TPR results, which indicate that there has been no reoxidation of the copper species under these reaction conditions.

**Figure 11:**
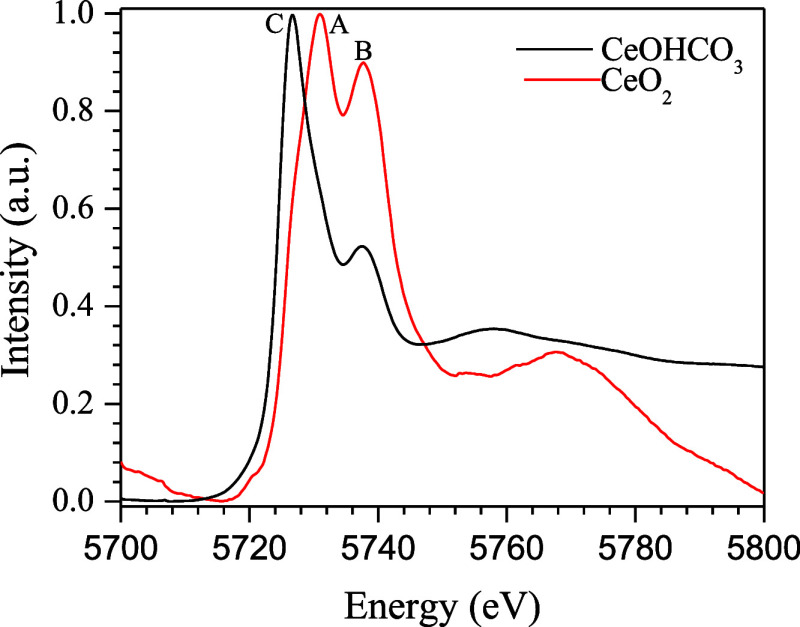
Ce LIII-edge XANES spectra of CeO_2_ and Ce(OH)CO_3_.

Figure 11 shows XANES spectra at the Ce LIII-edge during the reduction process of CeO_2_ and CeOHCO_3_, which are frequently used to quantify the Ce^4+^ and Ce^3+^ valence states.^43^ The CeO_2_ XANES spectrum exhibits two intense peaks, labeled A and B. Peak A, at 5731 eV, is attributed to absorption into the 4f level and is often utilized to gain a detailed understanding of the extent of ceria reduction. Peak B, at 5738 eV, corresponds to absorption into the 5d level, with no occupancy in the 4f level. The CeOHCO_3_ XANES spectrum displays one absorption maximum at 5725 eV, labeled as peak C, which is associated with 4f occupancy in the initial state.^44−46^

**Figure 12:**
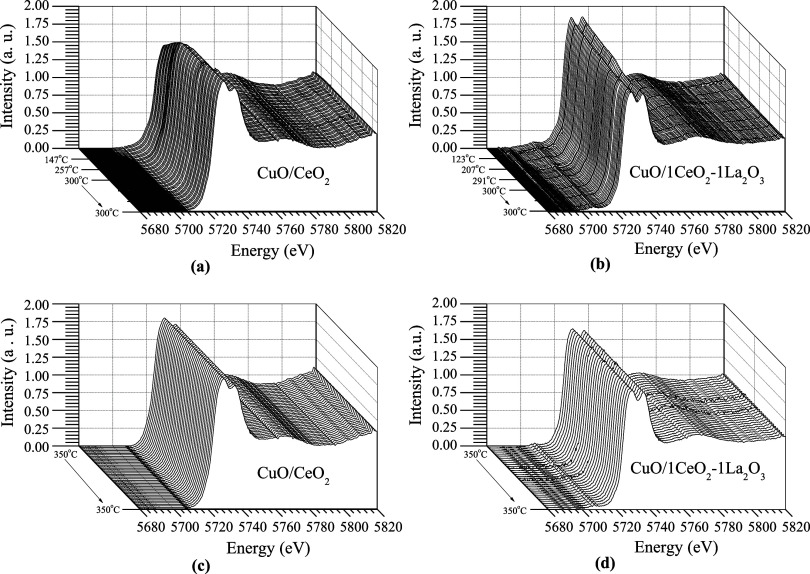
In situ XANES spectra at the Ce edge for the CuO/CeO_2_ and CuO/1CeO_2_-1La_2_O_3_ catalysts: (a and b) reduction process and (c and d) ideal WGS reaction.

In order to gain a better understanding of the variation in ceria oxidation states in the CuO/CeO_2_ and CuO/1CeO_2_-1La_2_O_3_ catalysts, in situ XANES measurements were performed at the Ce LIII-edge during the reduction process and ideal WGS reaction. Figure 12a,b shows XANES spectra at the Ce LIII-edge during the reduction process from room temperature to 300 °C for the CuO/CeO_2_ and CuO/1CeO_2_-1La_2_O_3_ catalysts, respectively. It can be seen that the CuO/CeO_2_ catalyst showed a small reduction of ceria; however, for the CuO/1CeO_2_-1La_2_O_3_ catalyst, it is not possible to observe changes from Ce^4+^ to Ce^3+^.

After the reduction, the furnace had been heated to 350 °C. Upon reaching this temperature, the reactor had been fed with reagents under the ideal WGS reaction conditions. Figure 12c,d shows the XANES spectra at the Ce LIII-edge during the real WGS reaction for the CuO/CeO_2_ and CuO/1CeO_2_-1La_2_O_3_ catalysts, respectively. In these conditions, the CuO/CeO_2_ catalyst had continued to show a small reduction of ceria, while the CuO/1CeO_2_-1La_2_O_3_ catalyst had continued to not exhibit any changes in the ceria oxidation state.

**Figure 13:**
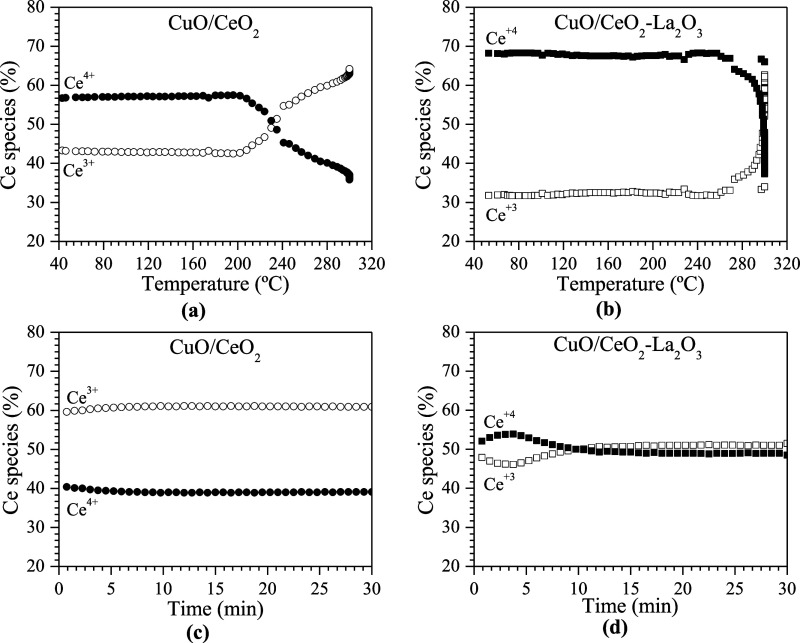
Quantification obtained from in situ XANES spectra at the Ce edge for the CuO/CeO_2_ and CuO/1CeO_2_-1La_2_O_3_ catalysts: (a and b) during the reduction process and (c and d) during the ideal WGS reaction.

To further elucidate the XANES results, Ce^3+^ and Ce^4+^ species were quantified by using a linear combination technique applied to the sample spectra. This method provided a clearer understanding of the changes in ceria oxidation states and helped to explain the observed variations in catalyst behavior. It is worth noting that this type of analysis has an error margin of approximately 10%. Figure 13a,b shows the results of this quantification for the reduction process, while Figure 13c,d shows the ideal WGS reaction for the CuO/CeO_2_ and CuO/1CeO_2_-1La_2_O_3_ catalysts. In Figure 13a, it can be observed that up to 196 °C the CuO/CeO_2_ catalyst contains approximately 57% Ce^4+^ and 43% Ce^3+^. Above this temperature, ceria reduction initiates. By 240 °C, the proportions of Ce^4+^ and Ce^3+^ become equal, and at 300 °C, their distribution is nearly reversed, with 40% Ce^4+^ and 60% Ce^3+^. As shown in Figure 13c, the amount of Ce^3+^ remains constant and is higher than that of Ce^4+^ until the end of the reaction. The CuO/1CeO_2_-1La_2_O_3_ catalyst exhibits behavior similar to that of the CuO/CeO_2_ catalyst. Up to 245 °C, the percentages of Ce^4+^ and Ce^3+^ are 70 and 30%, respectively. Above this temperature, ceria reduction begins, and at 300 °C, the quantities of the species balance out, each at 50%. During the first 8 min of reaction, as shown in Figure 13d, the percentage of Ce^4+^ increases to 54%, while Ce^3+^ decreases to 46%. After approximately 2 min, the quantities of Ce species initially equalized at 50% each, but shortly thereafter, Ce^3+^ species slightly exceeded Ce^4+^ species. Therefore, in the lanthanum-containing catalyst, Ce species are in a more oxidized state. This result was expected, as reported by other authors because the presence of La_2_O_3_ provides greater stability against the CeO_2_ transition phase, resulting in reduced sintering and minimized loss of metallic dispersion.^32^ Additionally, the presence of a trivalent element, such as La_2_O_3_, creates anionic vacancies in the CeO_2_ lattice, which enhances oxygen mobility and increases the catalyst oxygen storage capacity.^47,48^ This result is consistent with the XRD, TPR, and s-TPR results and specific surface area measurements after the reaction.

## 4 Conclusions

The study of the influence of La_2_O_3_ addition to CuO/CeO_2_ catalysts on the water–gas shift (WGS) reaction reveals several important findings regarding the catalytic performance and structural characteristics of these materials. XRD analysis confirms the formation of solid solutions with CeO_2_, leading to reduced crystallinity, lower specific surface area, and the creation of anion vacancies, which enhance oxygen mobility and storage capacity. These structural modifications improve the stability of La-doped catalysts, with CuO/1CeO_2_-1La_2_O_3_ demonstrating enhanced resistance to sintering and deactivation.

In terms of catalytic performance, CuO/CeO_2_ shows superior activity at lower temperatures, while catalysts with La, particularly CuO/1CeO_2_-1La_2_O_3_, perform better in the 250–350 °C range. However, CuO/2CeO_2_-1 La_2_O_3_ exhibits lower CO conversion due to the reduced surface area and metallic copper dispersion. Stability tests confirm that CuO/1CeO_2_-1La_2_O_3_ is more resistant to deactivation and sintering compared to non-La_2_O_3_ catalysts.

XANES spectra at the Cu edge indicate that the addition of La_2_O_3_ strengthens the interaction between CuO and the support, making the reduction of Cu^2+^ to Cu^0^ more challenging. Furthermore, La_2_O_3_ creates anion vacancies in the CeO_2_ lattice, improving oxygen mobility and enhancing structural stability.

Overall, while La_2_O_3_ addition improves catalyst stability and oxygen-related properties, its effect on catalytic activity is not consistently favorable. The optimization of the La_2_O_3_ content is necessary to achieve a balance between activity, stability, and structural robustness for effective application in the WGS reaction.
